# *Sparsorythussescarorum*, new species from Mindoro, Philippines (Ephemeroptera, Tricorythidae)

**DOI:** 10.3897/zookeys.795.28412

**Published:** 2018-11-05

**Authors:** Jhoana M. Garces, Ernst Bauernfeind, Hendrik Freitag

**Affiliations:** 1 Department of Biology, School of Science and Engineering, Ateneo de Manila University, Quezon City, Philippines Ateneo de Manila University Quezon City Philippines; 2 2nd Zoological Department (Entomology), Natural History Museum Vienna, Burgring 7, Vienna, Austria Natural History Museum Vienna Vienna Austria

**Keywords:** COI, Key Biodiversity Area, mayfly, Taugad River, taxonomy

## Abstract

A new mayfly species, *Sparsorythussescarorum***sp. n.** (Tricorythidae) is described from Mindoro Island, Philippines. Nymphs are characterized by the combination of the following characters: compound eyes of approximately equal size in both sexes, shape and setation of legs, presence of rudimentary gills on abdominal segment VII, and some details of mouthparts. Male imagines are characterized by the coloration pattern of wings and details of genitalia. The developmental stages are matched by DNA barcodes.

## Introduction

The order Ephemeroptera (mayflies) is a monophyletic group of pterygote hemimetabolous insects with aquatic larvae and delicate membranous wings in the adult stage. The presence of a subimaginal winged instar is unique within recent pterygote insects. Despite the notable organismic diversity in the Philippine Archipelago, only 38 species of mayflies (Insecta: Ephemeroptera) have been recorded so far. The last catalog by Hubbard and Pescador (1978) listed 20 species. New species and genera have been recorded afterwards from several parts of the Philippines by Flowers and Pescador (1984), and Müller-Liebenau (1980, 1982), with the most recent studies conducted by Braasch and Freitag (2008), Sroka and Soldán (2008), Braasch (2011) and Batucan et al. (2016). From these works, it can be inferred that there are eight families present in the Philippines: Baetidae, Caenidae, Ephemeridae, Heptageniidae, Leptophlebiidae, Prosopistomatidae, Teloganodidae and Tricorythidae. Some papers on mayflies of the country have been limited to ecological studies concerning mayfly nymphs (Realon 1979) and macroinvertebrate composition in certain freshwater bodies (e.g., Freitag 2004, Flores and Zafaralla 2012, Dacayana et al. 2013), albeit limited in number and scope as well. Nevertheless, the records regarding Philippines mayflies remain scattered and species diversity appears clearly underestimated.

In an effort to increase knowledge on the Philippine mayfly fauna, extensive sampling was done in Mindoro as part of the Baroc River Catchment Survey of the Ateneo de Manila University. The research group, as part of Bachelor of Science thesis, focused on the Key Biodiversity Area “69 Hinunduang Mt.”, classified as terrestrial and inland water area of very high biological importance and extremely high critical conservation priority.

A new species, *Sparsorythussescarorum* sp. n. belonging to the family Tricorythidae is described in this paper. The genus *Sparsorythus* Sroka & Soldán, 2008 (considered by Kluge 2010: 80 to represent a subgenus of *Tricorythus* Eaton, 1868) has been recorded from India, Indonesia, Sri Lanka, Vietnam and the Philippines, but is probably widespread in South and Southeast Asia. Listed below are the currently described species within the genus.

**Genus *Sparsorythus* Sroka & Soldán, 2008**

*Sparsorythusbifurcatus* Sroka & Soldán, 2008 (Vietnam)

*Sparsorythuscelebensis* (Kluge, 2010) (Indonesia: Sulawesi)

*Sparsorythusceylonicus* Sroka & Soldán, 2008 (Sri Lanka)

*Sparsorythusdongnai* Sroka & Soldán, 2008 (Vietnam)

*Sparsorythusgracilis* Sroka & Soldán, 2008 (India)

*Sparsorythusgrandis* Sroka & Soldán, 2008 (Indonesia: Java)

*Sparsorythusjacobsoni* (Ulmer, 1913) (Indonesia: Java, Sumatra; Sri Lanka; Philippines)

*Sparsorythusmultilabeculatus* Sroka & Soldán, 2008 (Vietnam)

*Sparsorythusbuntawensis* Batucan, Nuñeza & Lin (in Batucan et al.) 2016 (Philippines: Mindanao)

Aside from the recently described *Sparsorythusbuntawensis*Batucan et al., 2016 from Mindanao and the questionable record of *S.jacobsoni* (Ulmer, 1913) from Luzon (Ulmer 1924: 52), a female imago from Mindanao was reported as *Sparsorythus* sp. 4 by Sroka and Soldán (2008). A new species, *Sparsorythussescarorum* sp. n. from Taugad River, Mindoro Island, Philippines is described in this paper.

## Materials and methods

Nymphs were collected from rocks partially or fully submerged in the riffle section of the stream (Figs 10a–d). Winged specimens were attracted using a “black light” trap set-up from 6:30 PM to 8:00 PM under overcast skies near the streams or rivers. Insects were manually collected and stored in 96% ethanol to allow for genetic sequencing. Sample preparation for diagnosis under the dissecting microscope and compound microscope followed Braasch (2011) using Liquid de Faure (Adam and Czihak 1964) as mounting medium. Morphological examinations were performed using a Leica EZ4 stereo microscope and Olympus CX21 microscope. Processing and digital imaging of dissected parts was done using the latter stereo microscope equipped with DinoEye Eyepiece camera; the pictures were combined using CombineZP software (Hadley 2010) and were subsequently enhanced with Adobe Photoshop CS6. Full habitus photographs were taken under a Zeiss Axio Zoom V 16 microscope using diffuse LED lighting at magnifications up to 160×, with Canon 5D Mark II SLR attached to the microscope. Images were captured at various focus planes and subsequently stacked using the Zerene Stacker software. Morphological terminology followed Sroka and Soldán (2008) for nymph and imago, Koss and Edmunds (1974) for eggs and Edmunds and McCafferty (1988) for subimagines.

Specimens examined have been deposited in the following institutions:Museum of Natural History of the Philippine National Museum, Manila, Philippines (**PNM**); Ateneo de Manila University, Quezon City, Philippines (**AdMU**), Collection Jhoana Garces, Philippines (**CGM**), currently deposited in AdMU, and Museum für Naturkunde Berlin, Germany (**MNB**); and Naturhistorisches Museum Wien, Austria (**NMW**). Specimens at the latter repository are older and not collected by any of the authors, but they are presumably from the same locality.

Mitochondrial DNA extraction was done by elution with Qiagen DNeasy kit (Qiagen, Hilden, Germany) following the protocol for animal tissues (Qiagen 2002). For samples with successful DNA isolations, polymerase chain reactions (PCR) were performed using modified primers LC01490_mod (5’-TTTCAACAAACCATAAGGATATTGG-3’) and HC02198_mod (5’-TAAACTTCAGGATGRCCAAAAAATCA-3’) for amplification of a partition of the cytochrome c oxidase subunit (COI) gene. The PCR temperature progression was set: 180 s at 94 °C; 30 s at 94 °C, 30 s at 47 °C, 60 s at 72 °C (× 35 cycles); 300 s at 72 °C. Amplification success was checked by gel electrophoresis. PCR products of successful amplifications were sent to a commercial service for cleaning, cycle sequencing PCR and sequencing.

The sequences were manually traced and aligned using the software BIOEDIT version 7.2.5 (Hall 1999). Ends of each partition were trimmed to receive a complete matrix of all sequences used. The corresponding fragment of a COI sequence of *Sparsorythusgracilis* and *Sparsorythusbuntawensis* available from GenBank (Table 1; Batucan et al. 2016; Selvakumar et al. 2016) were included in the statistical parsimony analysis conducted with TCS 1.21 (Clement et al. 2000). The network connection limit was set manually to 1000 steps in order to keep sub-networks of different species connected and show their inter-specific genetic distance.

**Table 1.** ENA/GenBank accession numbers of DNA sequences, geographical origins, collection sites, and organismic sample references of specimens used for molecular-genetic analyses.

**Species**	**Locality**	**Code**	**Stage**	**Voucher**	**GenBank accession number**
*Sparsorythussescarorum* sp. n.	Mindoro	TR2L	Male Imago	EPH 2	MH595457
	Mindoro	HQCL	Female Subimago	EPH 42	MH595459
	Mindoro	HRCf	Nymph	EPH 43	MH595460
	Mindoro	369f	Nymph	EPH 5	MH595458
* Sparsorythus gracilis *	India				LC061853.1 ^1^
* Sparsorythus buntawensis *	Mindanao		Nymph	1.8.6	KT250142 ^2^

^1^Selvakumar et al. 2016; ^2^Batucan et al. 2016.

## Taxonomy

### Sparsorythus sescarorum sp. n.

Taxon classificationAnimaliaEphemeropteraTricorythidae

http://www.zoobank.org/961534FC-BA4E-43F4-ABB9-A7097BA70B31

Figures 1 , 2 , 3 , 4 , 5 , 6 , 7 , 8

#### Type locality.

Philippines, Oriental Mindoro, Municipality of Roxas, Barangay San Vicente: lower reach of Taugad River, a medium-sized mountain river and major tributary of the Baroc River, c. 12°37'18"N, 121°22'58"E, approximately 140 m asl (Figure 10c).

#### Type material.

**Holotype**: ♂ nymph (PNM), labelled“PHIL:Or.Mindoro, Roxas, Brgy. San Vicente, Taugad River; submerged rock surface, riffle; sec. veget.; c.12°37'18"N, 121°222'58"E, c.140m asl; leg. PS Cagande, J Garces, H Freitag 28.Nov.2017 (TR2g)M”, preserved in 95% ethanol, with complete set of gills and legs, one cercus partially broken near tip. **Paratypes**: 10 ♂ nymphs, same data as holotype [4 in MNB of which 1 on slide, 6 in CGM-AdMU of which 5 on slide]; 20 ♀ nymphs, same data as holotype [5 in PNM, 7 in MNB of which 1 on slide, 8 in CGM-AdMU of which 4 on slide]; 21 ♂ imagines, from exactly the same site as holotype collected using light trap on 28 Nov 2017 [7 in PNM, 4 in MNB of which 2 partly on slide, 10 in CGM-AdMU of which 6 partly on slide]; 24 ♀ subimagines, from exactly the same site as holotype collected using light trap on 28 Nov 2017 [8 in PNM, 5 in MNB of which 2 partly on slide, 11 in CGM-AdMU of which 2 partly on slide, 2 with corresponding eggs]; 1 ♂ subimago, from exactly same site as holotype, collected as nymph on 28 Nov 2017 and reared *in situ* in a mesh container [CGM-AdMU partly on slide]; 2 ♂ nymphs (NMW) labelled“Mindoro/ Mansalay/ Barok River 5km N Hinagdanan Fall/ coll. Mendoza 01-02-1995” [of which 1 on slide]; 2 ♀ nymphs (NMW) labelled as previous paratypes [of which 1 on slide]; 3 ♂ imagines (NMW) labelled as previous paratypes [of which 2 partly on slide]; 4 ♀ subimagines (NMW) labelled as previous paratypes [of which 1 partly on slide].

#### Description.

***Nymph*. ***Body* length 5.0–5.2 mm; ♂ cerci 0.8 and paracercus, 0.9 times body length; ♀ cerci and paracercus 0.9 times body length; head 1.9–2.0 times wider than long; antennae twice as long as head length (n = 10). General coloration of body brownish-yellow when preserved in alcohol.

**Figure 1.**
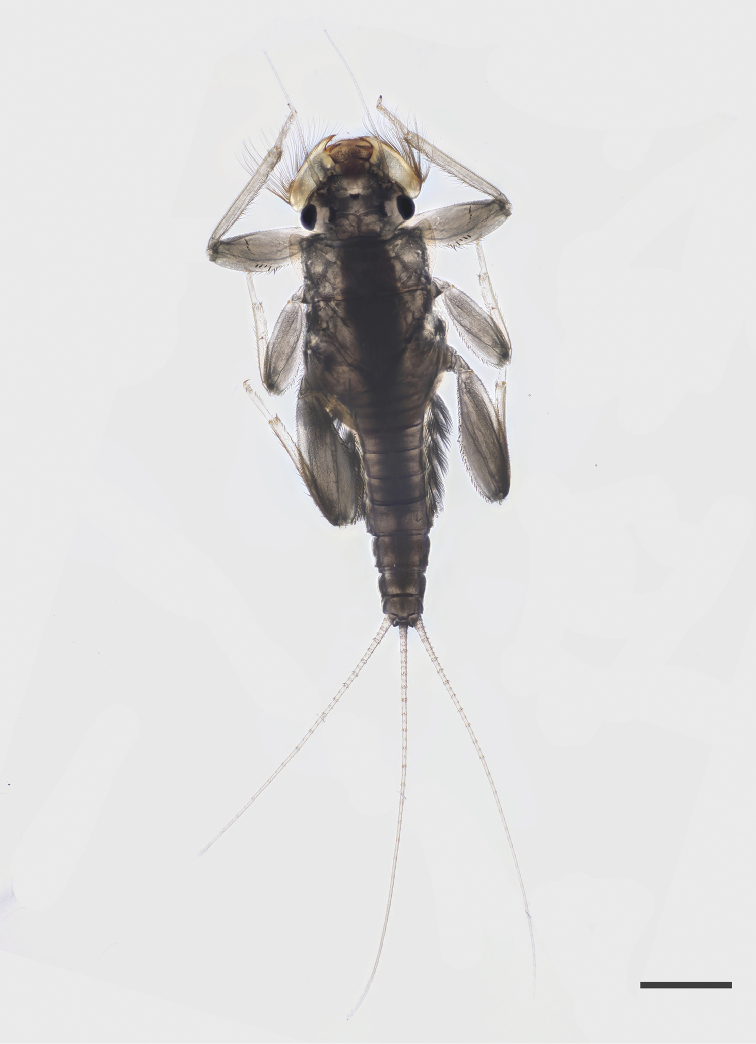
*Sparsorythussescarorum* sp. n., female nymph in dorsal view. Scale bar: 1.0 mm.

**Figure 2.**
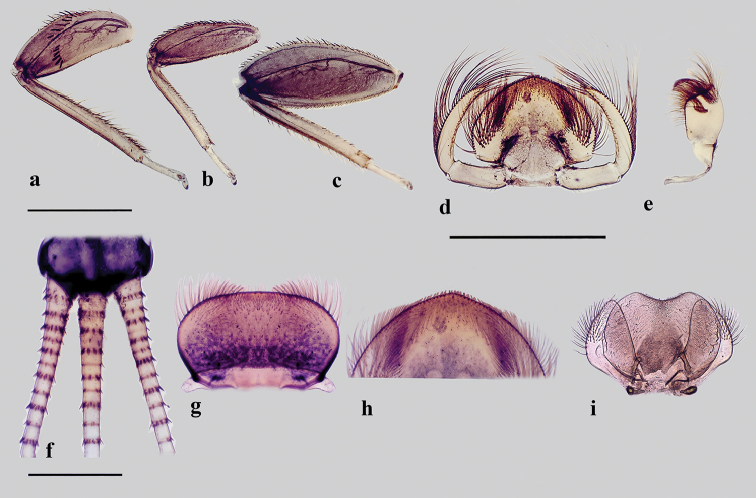
*Sparsorythussescarorum* sp. n., nymph. **a** fore leg **b** mid leg **c** hind leg **d** labium **e** maxilla **f** cerci and paracercus **g** labrum **h** labium anterior without apicomedial indentation **i** hypopharyngeal lingua. Scale bars: 1.0 mm (**a–c**); 1.5 mm (**d–e**); 0.5 mm (**f–i**).

**Figure 3.**
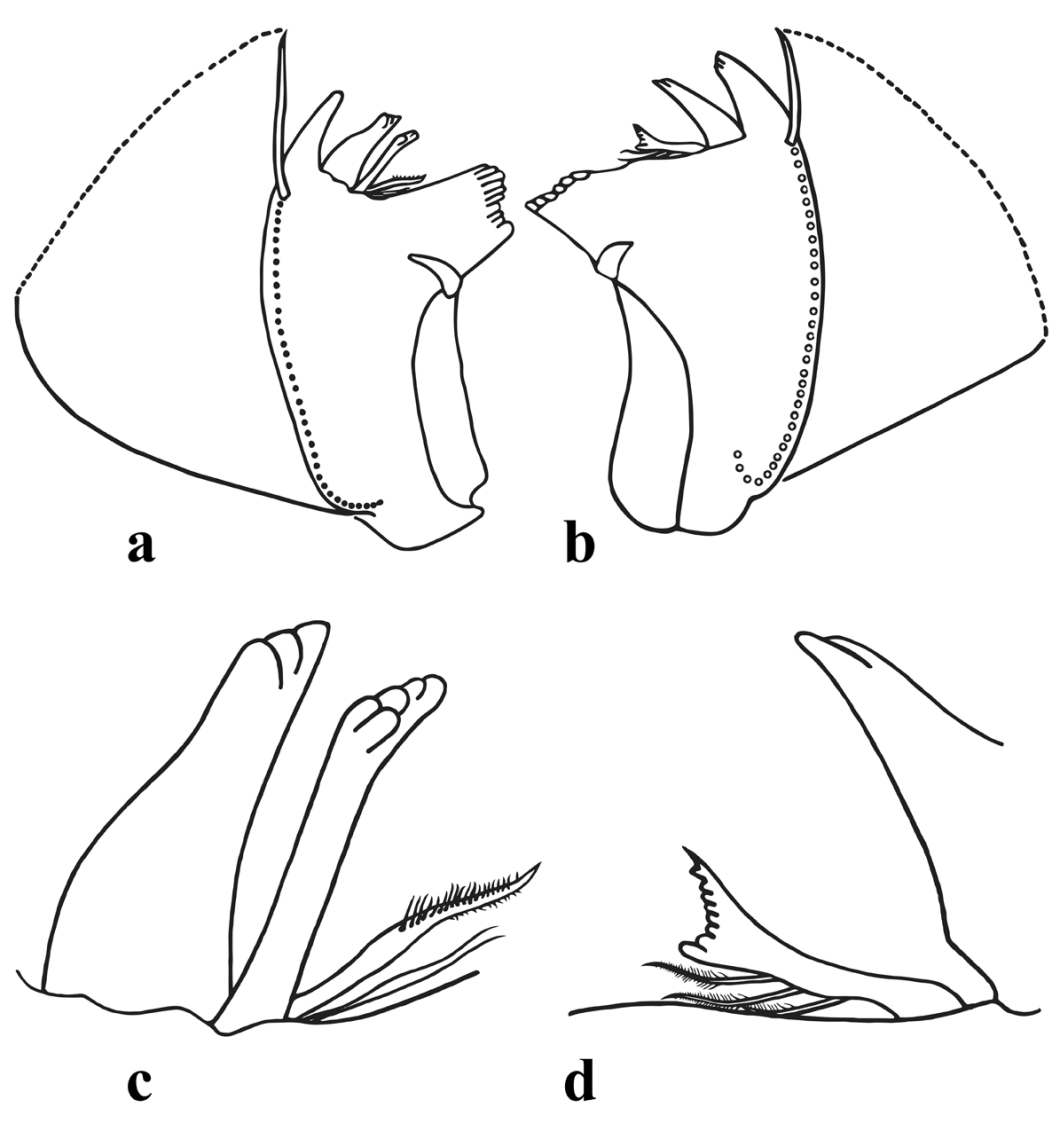
*Sparsorythussescarorum* sp. n., nymph. Mandibles and details **a** left mandible **b** right mandible **c** left prostheca **d** right prostheca.

*Head* (Figure 1) pale brownish-yellow. Male compound eyes blackish. Antenna yellowish, pedicle approx. 2.5 times longer than scape, surface of scape with almost transparent ribbon-shaped bristles, a few hair like setae and a finely chagrined area dorsally. Labrum (Figure 2g) oval; 2.8–3.0 times wider than long, with bristles medially diminishing in length along the anterior margin and laterally, uniformly scattered fine bristles on the dorsal surface. Two lateral groups of bristles on the ventral side. Hypopharyngeal lingua (Figure 2i) approximately as wide as long, with a short and shallow medio-longitudinal groove and wide apico-medial emargination; medial indentation relatively shallow, not exceeding 0.33 of hypopharyngeal lingua length, with uniformly scattered extremely small bristles; postero-lateral margin with 3–4 short, strong, evenly spaced bristles; superlingua rounded, bluntly pointed at apex, with a row of bristles in distal half of outer margin; bristles decreasing in length toward apex; inner margin of superlinguae straight (strongly concave in *S.buntawensis*). Mandibles (Figure 3a, b) as typical for the genus (Sroka and Soldán 2008); both outer incisors triangular; dorsal margin with numerous long filtering setae. Right prostheca (Figure 3d) 1/3 shorter than left, notched, expanded apically and bifurcate, with one long curved projection at distal part, bearing 3 finely fringed setae on the inner side. Distal part of left prostheca (Figure 3c) extended, with several short pointed teeth (blunt when worn); usually three long bristles (approximately ¾ of prostheca length) with feathery margins situated at base of prostheca (and frequently difficult to see). Maxilla (Figure 2e) oblong-shaped with truncate apex and anterolateral part with a group of strong bristles; a dense group of bristles medially and a regular oblique transversal row of slightly shorter bristles submarginally; maxillary palps absent; no sclerotized structures present. Labium (Figure 2d) with glossa and paraglossae fused into a rounded triangular plate; paraglossae with two groups of lateral submarginal bristles, the outer ones longer; labial plate without indentation or apico-medial incision (Figure 2h) (indentation present in *S.jacobsoni* sensu Ulmer 1939: Abb. 334); the whole plate surrounded by a regular row of setae diminishing apically in length; posterior margin of first segment of labial palp with 6 acutely pointed bristles.

**Figure 4.**
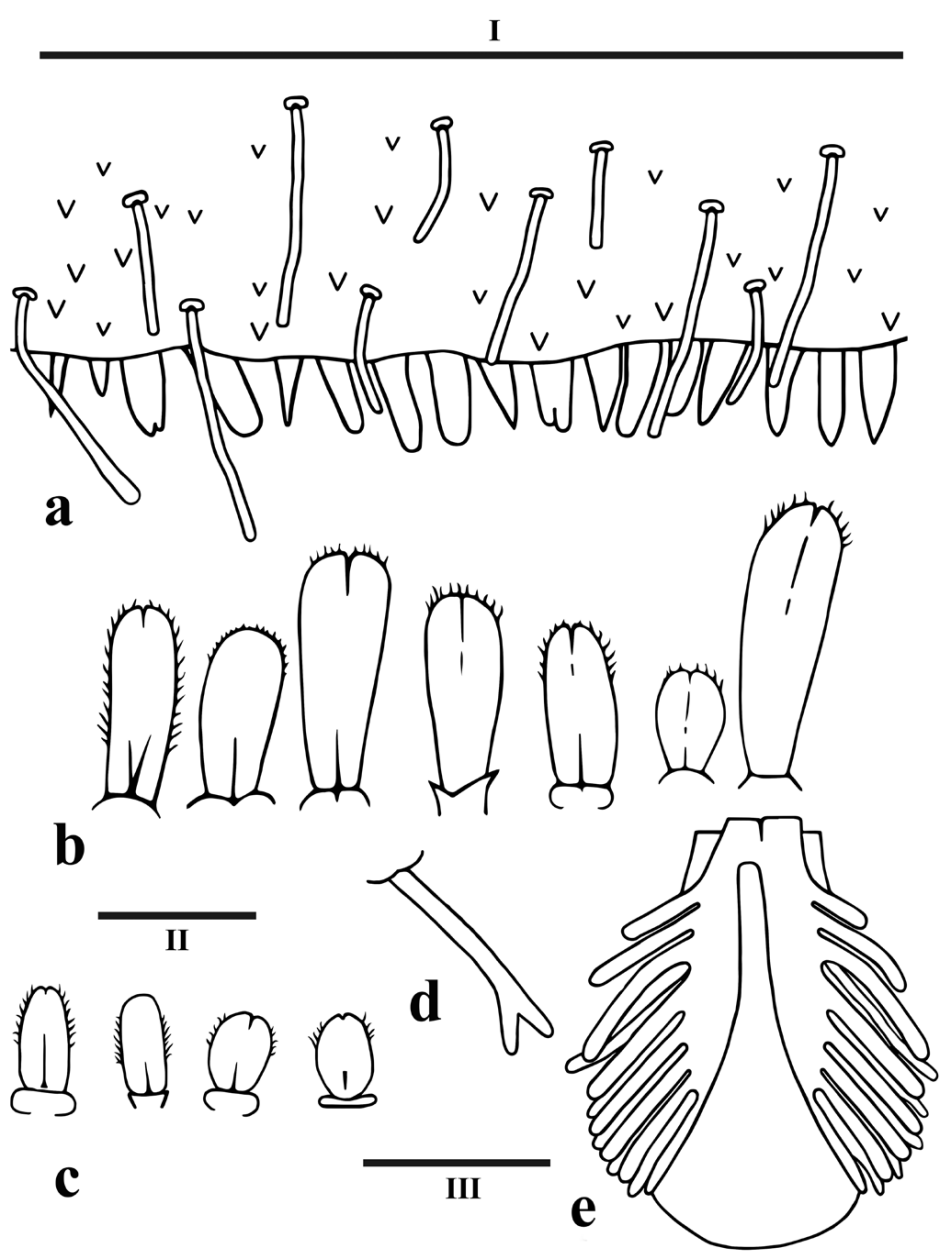
*Sparsorythussescarorum* sp. n., nymph. **a** 1 mm section of segment VII abdominal terga with small denticles and ribbon-shaped bristles **b** Fore femora transverse row of setae **c** hind femora irregularly scattered setae **d** gill VI **e** gill V. Scale bars: I – 1 mm (**a**); II – 0.1 mm (**b, c**); III – 0.25 mm (**d, e**).

*Thorax* (Figure 1) dorsally dull yellowish with blackish smudges and maculae, paler ventrally; pronotum laterally slightly enlarged with convex margins, distal margin more or less straight (in both sexes); wing pads dark, veins inconspicuous, in last instar larvae wing pads reaching the middle of abdominal segment II. Legs (Figures 2a–c) relatively robust; length ratio of femur : tibia : tarsus = 2.5 : 3.0 : 1.0 (fore legs), 2.5 : 2.5 : 1.0 (mid legs), 3.6 : 3.3 : 1.0 (hind legs). Fore femora (Figure 2a) flat, shorter than tibia; ratio length : width = 2.3 : 1.0; apically rounded strong spatulate bristles (Figure 4b), about 3.5–4.2 times longer than wide, arranged in a slightly irregular row almost perpendicularly crossing the femur, the row then abruptly bent basad and sinuously extending along the posterior margin of femur (somewhat similar to the “bow-shaped” arrangement in *S.ceylonicus* Sroka & Soldán, 2008); transverse row usually consisting of five bristles; the median part of the posterior margin with a scattered row of strong pointed bristles, anterior margin with a few bifid hair-like setae and submarginally a few almost transparent ribbon-shaped bristles; otherwise surface of femur glabrous, without setae or bristles. Fore tibiae with conspicuous inner submarginal row of apically pointed bristles, slightly longer than tibia width and a few (4–7) long marginal bristles. Fore tarsus with a row of 6–10 strong pointed bristles along the inner margin and a few irregularly scattered bifid setae. Surface of middle and hind femora sparsely covered with stout spatulate bristles (Figures 4c, e) one-third of marginal bristle size and fine ribbon-shaped bristles. Middle femora (Figure 2b) with a dense row of blunt, slender spatulate (rarely pointed) bristles along the dorsal (posterior) margin, the basal half of posterior margin submarginally with some small spatulate bristles; ventral margin with a scanty row of medium sized blunt or slender spatulate bristles, more numerous and slightly longer in basal part; surface of femur with some very small oval bristles and fine transparent ribbon-shaped bristles, the latter more numerous submarginally. Middle tibiae with an inner submarginal row of apically bluntly pointed bristles, about ½ of tibia width, outer margin with about a dozen long pointed bristles and scattered bifid setae. Hind femora (Figure 2c) with a dense row of blunt, slender spatulate (rarely pointed) bristles along the dorsal (posterior) margin, ventral (anterior) margin with several rows of distinctly smaller, slender spatulate and oval shaped bristles. Surface with scattered small oval bristles and fine ribbon-shaped bristles. Hind tibiae with inner marginal row of slender spatulate bristles, almost as long as bristles along posterior margin of femur; outer margin of tibia with a dense row of long, bluntly pointed bristles, interspersed with acutely pointed bristles (with finely feathery margins), scattered bifid setae and long hair-like setae (especially in distal half). Claws strongly hooked, with 2–3 teeth and a pair of strong pointed processes approximately in the middle. Dark tracheization conspicuous on all femora.

*Abdominal terga* (Figure 1) brownish with fine darker stippling, a small light medial dot and two pale yellowish brown paramedial patches; posterior part of terga VIII and IX darker; terga darker than sterna with greyish-black stippling; segments II–VII with gills. Gills on segments II–VI similar in shape (Figure 4e) and diminishing in size, each consisting of a dorsal ellipsoidal plate and two branched ventral membranous parts with dense filaments; gill plate on segment II reaches middle of abdominal segment IV, gill plate on segment VI reaches almost end of abdominal segment VII; gill plates simple, thin, not enforced, with scattered hair-like marginal bristles; rudimentary gill on segment VII (Figure 4d) small, tubular with bifurcate tip and frequently missing (or lost subsequent to collecting), without plate. Surface of terga with small denticles and ribbon-shaped bristles, the latter more densely distributed in lateral parts and a few scattered hair-like setae; posterior margin of terga (Figure 4a) with rather tongue-shaped teeth, acutely pointed, blunt or with somewhat frayed tips (worn). Abdominal terga without postero-lateral processes. Abdominal sterna with a few narrow ribbon shaped bristles in posterior lateral area, hind margin of sterna smooth. Posterior margin of sternum IX equally shaped in male and female larvae.

*Paracercus* (Figure 1) in male nymphs usually slightly longer than cerci, subequal in female nymphs; surface of segments without bristles; posterior margin of segments with strong, slender spatulate or bluntly pointed bristles of approx. ½ (basal segments) to ^1^/_3_ of segment length (Figure 2f), tips of bristles extremely finely frayed. Sexual dimorphism in the spatial arrangement and width of cerci: ♂ with basal segments of cerci and paracercus broader and continuous; ♀ basal segments of cerci and paracercus distinctly more slender and not touching.

**Figure 5.**
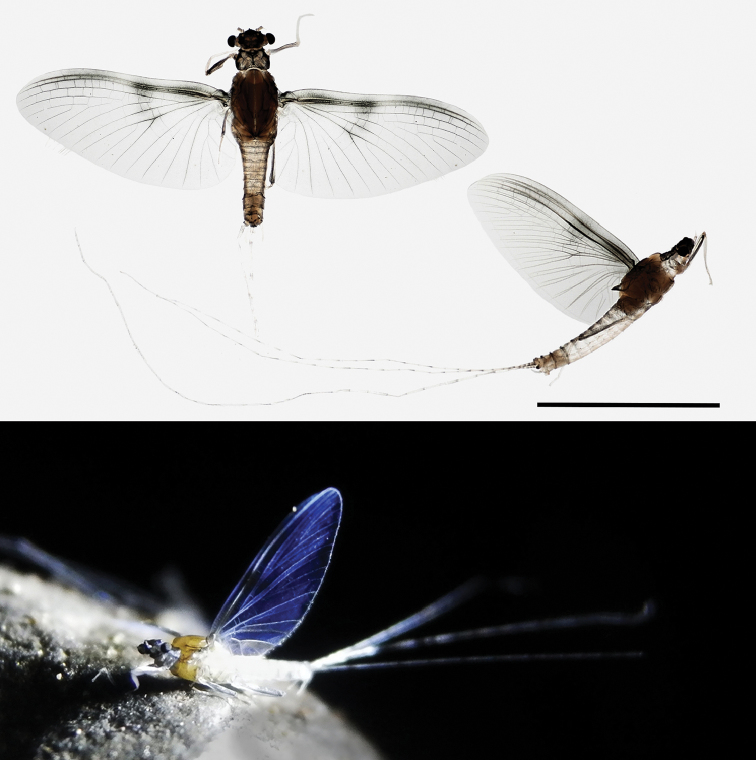
*Sparsorythussescarorum* sp. n., male imago. Scale bar: 4.0 mm.

***Male imago.****Body* length 4.5–4.8 mm; fore wing 4.0–4.5 mm; antenna 1.2 mm long; tibia 1.0 mm; cerci and paracercus length approx. 10–12 mm. General color of head and prothorax dark, blackish (Figure 5); antennal pedicle and posterior margin of eyes paler; mesothorax pale yellowish brown; abdomen white to pale greyish with black stippling and maculation on posterior margin; ventral thorax and abdomen paler, whitish and more transparent than dorsal side; tracheization not pigmented; cerci white to pale greyish, at least basal segments frequently with narrow black posterior border; forceps whitish to transparent; legs pale greyish, femora darker, finely stippled with black along margins. Fore wings transparent with minimal dark grey smudges in basal half; most dark smudges in the costal and subcostalareas, clustered in basal and apical regions; pterostigmatic region milky, usually no cross veins in costal space discernible; venation mostly whitish, black in the center of the wing, almost transparent towards the margins; veins costa, subcosta and radius anterior rather transparent, broadly bordered with intense black stippling and conspicuous over all their length. Intensity of dark stippling on body, legs, and wings varies considerably between individuals.

**Figure 6.**
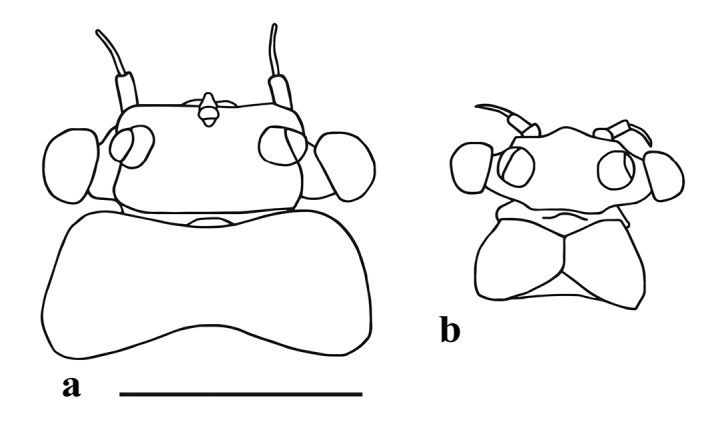
Head and prothorax of *Sparsorythussescarorum* sp. n.: **a** female subimago **b** male imago. Scale bar: 1 mm.

*Head* (Figure 6b) with globular compound eyes, of approximately the same size as in females, distanced approximately half of mesothorax width; antennal pedicle approximately 2.5 times longer than scape. Prothorax (Figure 6b) slightly longer than head. Tarsal claws double on all legs; fore legs with two rounded claws, mid and hind legs with one claw rounded and the other pointed (ephemeroid). Femur slightly longer than tibia, length ratio 1.2 : 1. In the fore wing vein media forked at approximately ½ of its length; veins cubitus posterior and analis frequently not visible along their entire length, transparent in apical part; posterior wing margin with fine setae, more scattered distally.

**Figure 7.**
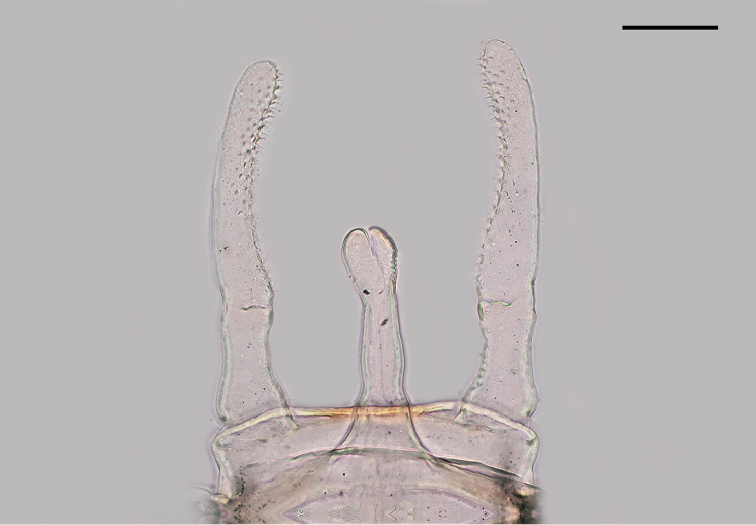
*Sparsorythussescarorum* sp. n., male genitalia (imago). Scale bar: 0.1 mm.

*Genitalia* (Figure 7) with subgenital plate entire. Forceps two-segmented; basal segment shorter than distal one, length ratio approximately 1.0 : 2.2; forceps segment I cylindrical, widest at base, slightly constricted in the middle; hind margin of forceps base sclerotized in medial part with a few tiny bristles; inner margin of segment two of forceps covered with numerous leaf-shaped attachment structures. Penis lobes simple, straight and tubular, slightly bent in dorsal direction, only slightly constricted subapically; penis apex reaching approximately the basal quarter of second forceps segment; apex of penis rounded with distinct medial emargination bisecting penal apex. Caudal filaments more than twice the body length, approx. 10–12 mm, cerci glabrous but paracercus sparsely covered with fine setae.

***Male subimago.*** Similar to imago, but wings uniformly greyish and with microtrichae on wing surface; tarsus of fore leg with one pointed and one obtuse claw (= ‘ephemeropteroid’ sensu Kluge 2004: 34, Kluge 2010); fore femur slightly shorter than tibia, length ratio 0.9 : 1.0; cerci and paracercus longer than body, but distinctly shorter than in imago. Male genitalia almost as in imago, but forceps segment I stouter.

**Figure 8.**
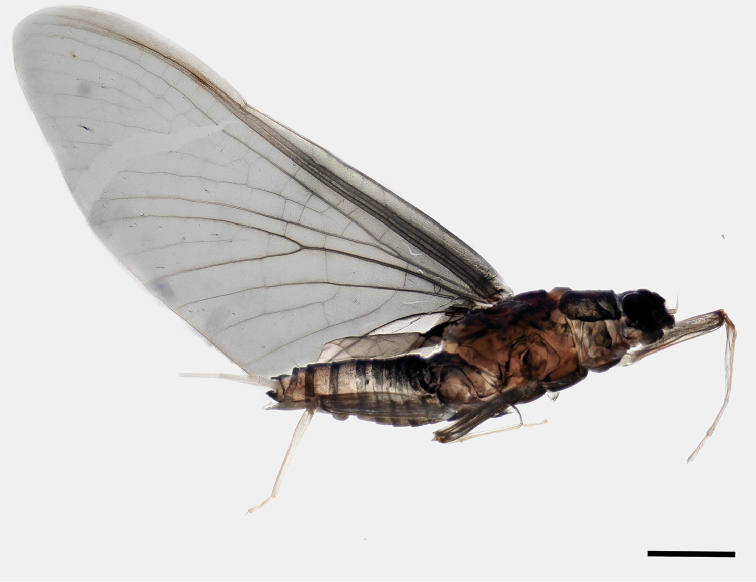
*Sparsorythussescarorum* sp. n., female subimago. Scale bar: 1 mm.

***Female subimago.****Body* length 4.0–4.6 mm; fore wing 5.0–5.2 mm; cerci and paracercus length 3.5–4.0 mm. General coloration of head, prothorax, dorsal mesothorax and dorsal abdomen dark, brownish or blackish (Figure 8); ventral mesothorax yellowish brown; cerci whitish, densely covered with long setae. Head (Figure 6a) with globular compound eyes, of approximately the same size as in male imagines, distanced approximately half of mesothorax width; antennal pedicle approximately 2.5 times longer than scape. Femora blackish, basal end of fore femur paler than the rest, tibia and tarsus transparent. Tarsal claws double on all legs, one rounded and the other pointed (ephemeroid). Length ratio femur: tibia: tarsus = 3.0: 3.2: 1.0 (fore legs), 3.1: 3.0: 1.0 (middle legs), 4.8: 4.1: 1.0 (hind legs). Fore wings (Figure 8) gray with dark smudges in basal half; most dark smudges in the costal and subcostal space clustered in two regions; veins costa and subcosta distinctly darker and conspicuous over all their length; longitudinal venation darker anteriorly and proximally. Subimaginal falciform microtrichia present on wing surface, body surface, and legs. Outer and inner edges of wings (wing margin) with a seam of long and fine setae, slightly shorter towards the wing tip. Subanal plate (sternum IX) approximately as wide at base as long, smoothly rounded in distal half and more than one third longer than sternum VIII (compare Sroka and Soldán 2008: fig. 64).

**Eggs.** Approximately 190 × 120 μm, epithema (polar cap) covering approximately ⅕ of total egg length. Surface smooth, covered by typical shallow polygonal ridges (almost identical to Sroka and Soldán 2008: fig. 72). Micropyle very small, tagenoform.

**Figure 9.**
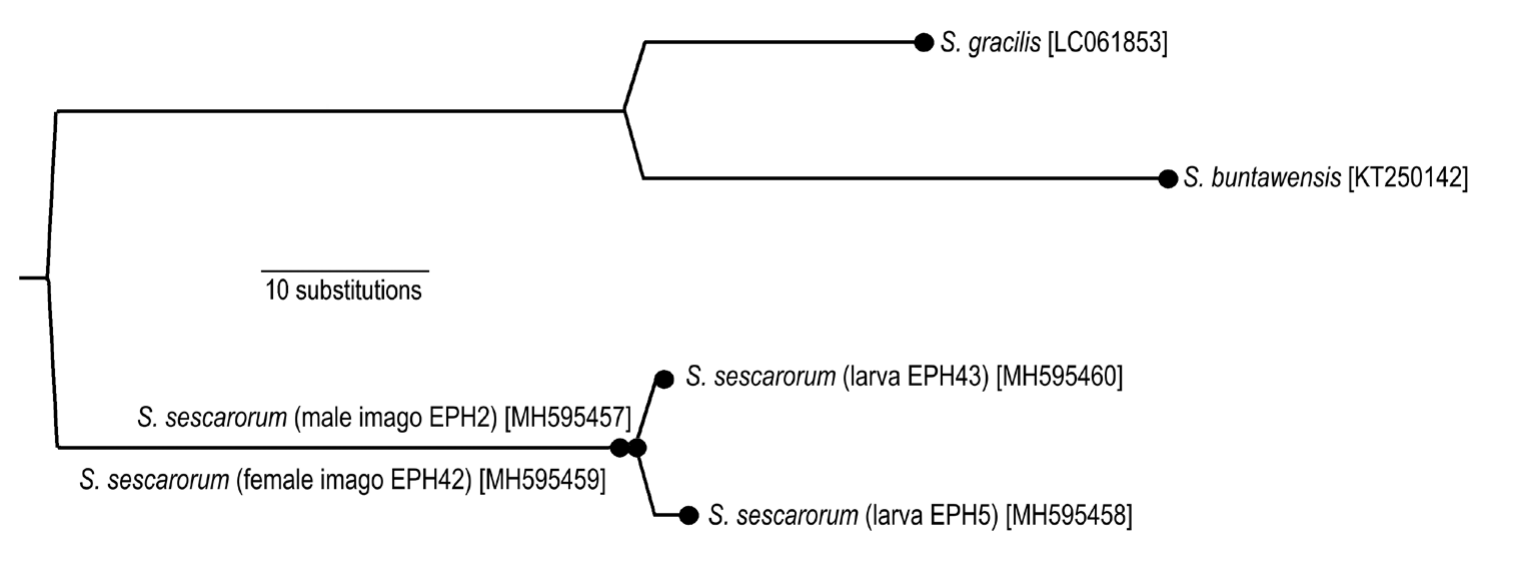
Statistical parsimony haplotype network of successfully sequenced samples, *Sparsorythusgracilis* and *Sparsorythusbuntawensis* sequences from GenBank from aligned COI sequences of 523 bp. Filled circles represent haplotypes as labelled.

The resulting network tree (Figure 9) demonstrates that the conspecific specimens of different life stages of *Sparsorythussescarorum* sp. n. have only a maximum of five substitutions compared to the much higher divergence of the other *Sparsorythus* species sampled. This Statistical Parsimony tree is solely intended to provide evidence for matching larval and imaginal stages.

#### Differential diagnosis.

The nymph of *Sparsorythussescarorum* sp. n. differs from all known Oriental tricorythid taxa in the combination of the following characters: apex of hypopharyngeal lingua with wide medial indentation (similar in *S.buntawensis*), wing pads reaching the middle of abdominal segment II in last instar larvae, hind femora longer than tibia (length ratio of femur : tibia : tarsus = 3.6 : 3.3 : 1.0) with central femur surface glabrous (only a few tiny bristles submarginally) and bifurcate rudimentary gill on segment VII present. The new taxon in some respects somewhat resembles *S.bifurcatus* and *S.gracilis*, but leg ratio of hind femur : tibia : tarsus and setation of femora are distinctive. Unlike *S.jacobsoni* (sensu Ulmer 1939: Abb. 334), *S.sescarorum* has no small nick in the median anterior margin of its labial plate and possesses a specifically shaped transverse row of setae on fore femora, and the rudimentary gill is bifurcate instead of filamentous. Unlike *S.buntawensis, S.sescarorum* sp. n. has inner margin of superlinguae straight, bifurcate rudimentary gill and cerci and paracercus shorter than body length. They can be easily differentiated using leg ratios of femur : tibia : tarsus and fore femora length : width. The arrangement of apically rounded setae on fore femur resembles the bow-shaped arrangement of *S.ceylonicus*.

Male genitalia are comparatively similar within the genus *Sparsorythus*. The male imago of *Sparsorythussescarorum* sp. n. can be differentiated from other Oriental tricorythid taxa based on the pattern of dark smudges in the fore wing, the medial sclerotization along the hind margin of forceps base and the length ratio of forceps segments. Color pattern of wings is rather similar in *S.multilabeculatus*, but male imagines of *S.sescarorum* are significantly larger (4.5–4.8 mm vs. 3 mm in *S.multilabeculatus*). Male imagines of *S.sescarorum* have globular compound eyes, of approximately the same size as in females, in contrast to *S.bifurcatus* and *S.dongnai* compound eyes which are distinctly larger than in females. Identification of female subimagines remains rather difficult (except by direct comparison of specimens), mainly based on coloration, color pattern of wings, length ratio of legs, shape of subanal plate (sternum IX) and exochorionic structures of eggs.

#### Distribution.

The species is so far only known from the type locality, lower reach of Taugad River, Oriental Mindoro, Philippines.

#### Ecology.

**Figure 10.**
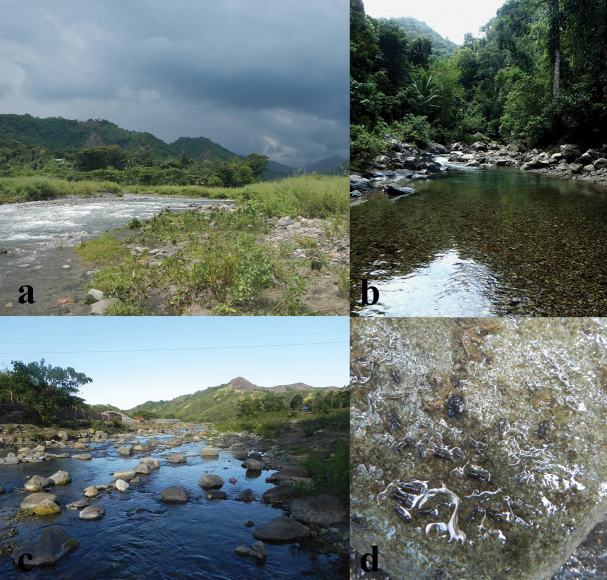
Collection sites of *Sparsorythussescarorum* sp. n. in Roxas, Oriental Mindoro: **a** lower Hinundugan River, a tributary of the Baroc River **b** upper Hinundugan River **c** type locality, lower reach of Taugad River, a major tributary of the Baroc River **d** submerged rocks with nymphs (lifted above water surface), the typical larval habitat.

All material was collected from or near permanent rivers in Oriental Mindoro. This province has an equatorial monsoonal (Am) climate based on the Köppen-Geiger Classification and is nationally recognized as the Type III climate according to the Modified Corona Classification (Kintanar 1984), characterized by absence of a very pronounced maximum rain period and a short dry season, in Oriental Mindoro during the period of February to April. Average temperature is around 27.4 °C and the average annual rainfall about 2000 mm (PAGASA 2018), however with considerable annual and local variations. All collection sites are at low altitudes of 5–250 m a.s.l. at meandering alluvial rivers of small to medium size (2–12 m wide) comparable to the hyporhithral section (Figures 10a–c) with estimated water discharge ranging from 0.006 to 7.0 m³/s during the respective times of collection. Most of these sites were surrounded by secondary vegetation, rarely secondary forest, with few houses and farmland in some distance from the river bed.

Larvae were collected in lotic river sections at water depth ranging from 3 to 35 cm, predominantly from mineral bottom substrates (typically small to medium-sized boulders in riffles (Figure 10d)), rarely from submerged wood. The water currents at these microhabitats were estimated to range from 0.08 to 0.79 m/s (usually ca. 0.2–0.4 m/s). The temperature of the water ranged from 23.0 to 28.7 °C, the pH from 6.8 to 8.3, dissolved oxygen from 3.8 to 8.3 mg/l (mostly, but not always near 100% saturation), biochemical oxygen demand (BOD_5_) from 0.1 to 1.3 mg/l. The maximum values, respectively, measured for selected dissolved nutrients were as follows: phosphate 0.7 mg/l, ammonium 0.5 mg/l, nitrate 1.0 mg/l. Dissolved nitrites were always below detectable values (< 0.2 mg/l). Imagines and subimagines were collected from light traps placed along the same river sections. They seemed to be most attracted by black light used at a time shortly after sun set. No information on feeding, type of emergence and life cycle is available at present. Presumably subimagines emerge on the water surface and male subimagines moult almost immediately after emergence whereas females retain the subimaginal stage.

#### Etymology.

The name of this new species is given to acknowledge the efforts of Baranggay Captain Ronel S. Sescar, Baranggay Kagawad for Environmental and Agriculture concerns Rodel S. Sescar and the rest of their family members who were instrumental for the protection and preservation of the Baroc River. Assessments of aquatic biodiversity and training of student researchers would not have been possible without their support for the past few years.

## Discussion

Sroka and Soldán (2008) revised the hitherto known Tricorythidae from the Oriental Region, restricting the genus *Tricorythus* Eaton, 1868 to the Afrotropical Region and proposing the new genus *Sparsorythus* (type species *Sparsorythusbifurcatus* Sroka & Soldán, 2008) for Oriental tricorythid taxa. Kluge (2010) redescribed *Tricorythusvaricauda* Pictet, 1843 (type species of *Tricorythus*) recognizing *Madecassorythus* Elouard & Oliarinony, 1997, *Spinirythus* Oliarinony & Elouard in Oliarinony et al., 1998, *Ranorythus* Oliarinony & Elouard, 1997 and *Sparsorythus* Sroka & Soldán, 2008 as subgenera of *Tricorythus*. Lineages within Afrotropical *Tricorythus*, however, are still poorly known (Barber-James 2008) and for the present the opinion of Sroka and Soldán (2008) is followed in this paper.

Several characters of *Sparsorythussescarorum* sp. n. merit comment. The nymphs of *S.sescarorum* sp. n. exhibit a sexual dimorphism in the spatial arrangement and width of cerci and paracercus as observed in other Tricorythidae. Size of eyes is about equal in male and female specimens in the larval and winged stages, whereas *S.bifurcatus* and *S.dongnai* exhibit distinctly larger eyes in male specimens. Kluge (2010) suggested that some species of *Tricorythus*, such as *T.exophthalmos*, show a correlation between enlarged male eyes and the sexually dimorphic shape of the pronotum, where the male pronotal fore margin expands medially forming a semicircular flap that overlaps the hind part of the head, while the female fore margin is straight. The fore margin of *S.sescarorum* sp. n. larval pronotum is more or less straight in both sexes, lending some support to the opinion of Kluge.

Female adults obviously retain the subimaginal stage. This has also been observed at least in *Tricorythusvaricauda*, *Sparsorythuscelebensis*, and some other tricorythid taxa (Kluge 2010). Male subimagines of the new species have never been collected at light traps, however a single specimen from the type locality is available which has been obtained by rearing nymphs and which obviously represents a subimago. This suggests that the subimaginal-imaginal molting of males occurs immediately after emergence before the first flight.

## Supplementary Material

XML Treatment for Sparsorythus sescarorum

## References

[B1] Adam H Czihak G (1964) Arbeitsmethoden: der makroskopischen und mikroskopischen Anatomie. G. Fischer, Stuttgart, 583 pp.

[B2] Barber-James HM (2008) A synopsis of the Afrotropical Tricorythidae. In: Hauer FR Stanford JA Newell RL (Eds) International advances in the ecology, zoogeography and systematics of mayflies and stoneflies.University of California Publications in Entomology 128 [Proceedings of the 11^th^ International Conference on Ephemeroptera and the 15^th^ International Symposium on Plecoptera, Montana, USA, 22–29 August 2004], 187–203. 10.1525/california/9780520098688.003.0014

[B3] Batucan LS Nuneza OM Villanueva RJT Lin CP (2016) A new species of mayfly (Ephemeroptera: Tricorythidae) from Mindanao Island, Philippines and association of life stages using DNA barcodes. Philippine Journal of Systematic Biology 10: 6–13.

[B4] Braasch D (2011) New species of the family Heptageniidae (Ephemeroptera) from Borneo and the Philippines. Deutsche Entomologische Zeitschrift 58(2): 201–219. 10.1002/mmnd.201100024

[B5] Braasch D Freitag H (2008) *Palawaneuria*, a new subgenus of *Compsoneuria* and new species of *Compsoneuria* and *Afronurus* (Ephemeroptera, Heptageniidae) from Palawan, Philippines. Deutsche Entomologische Zeitschrift 55(1): 117–128. doi/10.1002/mmnd.200800009.

[B6] Clement M Posada D Crandall KA (2000) TCS: a computer program to estimate gene genealogies. Molecular Ecology 9: 1657–1659. 10.1046/j.1365-294x.2000.01020.x 11050560

[B7] Dacayana CML Hingco JT Del Socorro MML (2013) Benthic macroinvertebrate assemblage in bulod river, Lanao del Norte, Philippines. Journal of Multidisciplinary Studies 2(1): 398. 10.7828/jmds.v2i1.398

[B8] Eaton AE (1868) An outline of the re-arrangement of the genera of Ephemeridae. Entomologist’s Monthly Magazine 5: 82–91.

[B9] Edmunds GF jr McCafferty WP (1988) The Mayfly Subimago. Annual Review of Entomology 33: 509–529. 10.1146/annurev.en.33.010188.002453

[B10] Elouard J-M Oliarinony R (1997) Biodiversité aquatique de Madagascar: 6. *Madecassorythus* un nouveau genre de Tricorythidae définissant la nouvelle sous-famille des Madecassorythinae (Ephemeroptera, Pannota). Bulletin de la Société entomologique de France 102(3): 225–232.

[B11] Flores MJL Zafaralla MT (2012) Macroinvertebrate composition, diversity and richness in relation to the water quality status of mananga river, Cebu, Philippines. Philippine Science Letters 5(2): 103–113.

[B12] Flowers RW Pescador ML (1984) A new *Afronurus* (Ephemeroptera: Heptageniidae) from the Philippines. International Journal of Entomology 26: 362–365.

[B13] Freitag H (2004) Composition and Longitudinal Patterns of Aquatic Insects Emergence in Small Rivers of Palawan Island, the Philippines. International Review of Hydrobiology 89(4): 375–391. 10.1002/iroh.200310710

[B14] Hadley A (2010) CombineZP. http://www.hadleyweb.pwp.blueyonder.co.uk/CZP/News.htm [Version of 6 June 2010]

[B15] Hall TA (1999) BioEdit: a user-friendly biological sequence alignment editor and analysis program for Windows 95/98/NT. Nucleic Acids Symposium Series 4: 95–98.

[B16] Hubbard MD Pescador ML (1978) A catalog of the Ephemeroptera of the Philippines. Pacific Insects 19: 91–99.

[B17] Kintanar RL (1984) Climate of the Philippines, PAGASA report, Manila, 38 pp.

[B18] Kluge N (2004) The phylogenetic system of Ephemeroptera. Kluwer Academic Publishers, Dordrecht, 456 pp 10.1007/978-94-007-0872-3

[B19] Kluge N (2010) Redescription of the taxon Tricorygnatha (Ephemeroptera, *Tricorythus* s.l.) based on new finding in Africa and Indonesia. Russian Entomological Journal 19(2): 79–104.

[B20] Koss RW Edmunds GF Jr (1974) Ephemeroptera eggs and their contribution to phylogenetic studies of the order. Zoological Journal of the Linnean Society 55: 267–349. 10.1111/j.1096-3642.1974.tb01648.x

[B21] Müller-Liebenau I (1980) *Jubabaetis* gen.n. and *Platybaetis* gen.n., two new genera of the family Baetidae from the Oriental Region. In: Flannagan JF Marshall KE (Eds) Advances in Ephemeroptera Biology.Plenum, New York, 103–114. 10.1007/978-1-4613-3066-0_8

[B22] Müller-Liebenau I (1982) New species of the family Baetidae from the Philippines (Insecta, Ephemeroptera). Archiv für Hydrobiologie 94(1): 70–82.

[B23] Oliarinony R Elouard J-M Raberiaka NH (1998) Biodiversité aquatique de Madagascar. 8. *Spinirythus* un nouveau genre de Tricorythidae (EphemeropteraPannota). Bulletin de la Société entomologique de France 103(3): 237–244.

[B24] PAGASA [Philippine Atmospheric, Geophysical and Astronomical Services Administration] (2018) Monthly rainfall, by Station, Year, and Month. http://philfsis.psa.gov.ph/index.php/id/15/matrix/J20FSMRI

[B25] Pictet FJ (1843) Histoire naturelle générale et particulière des insects névroptères, Famillie des éphémérines. J. Kessmann [et A. Cherbuliez], Genève, 319 pp.

[B26] Qiagen (2002) DNeasy Tissue Kit Handbook 05/2002. Hilden, Germany, 43 pp.

[B27] Realon CBR (1979) An ecological study of mayfly nymphs I Molawin Creek, Mt. Makiling, Laguna. The Philippine Entomologist 4(4): 233–291.

[B28] Selvakumar C Sivaramakrishnan KG Janarthanan S (2016) DNA barcoding of mayflies (Insecta: Ephemeroptera) from South India. Mitochondrial DNA Part B 1: 1, 651–655. 10.1080/23802359.2016.1219623 PMC7800591 33473584

[B29] Sroka P Soldán T (2008) The Tricorythidae of the Oriental Region. International Advances in the Ecology, Zoogeography and Systematics of Mayflies and Stoneflies. University of California Press, Oakland 128, 313–354. 10.1525/california/9780520098688.003.0021

[B30] Ulmer G (1913) Ephemeriden aus Java, gesammelt von Edw. Jacobson. Notes from the Leyden Museum 35: 102–120.

[B31] Ulmer G (1924) Ephemeropteren von den Sunda-Inseln und den Philippinen. Treubia 6: 28–91.

[B32] Ulmer G (1939) Eintagsfliegen (Ephemeropteren) von den Sunda-Inseln. Archiv für Hydrobiologie, Supplement 16: 443–692.

